# What’s new in insomnia? Diagnosis and treatment

**DOI:** 10.1590/0004-282X-ANP-2022-S124

**Published:** 2022-08-12

**Authors:** Álvaro Pentagna, Luiz Henrique Martins Castro, Bárbara Araújo Conway

**Affiliations:** 1 Universidade de São Paulo, Faculdade de Medicina, Hospital das Clínicas, Divisão de Clínica Neurológica, Ambulatório de Medicina do Sono, São Paulo, SP, Brazil.; 2 Universidade de São Paulo, Faculdade de Medicina, Curso de Pós-Graduação, Departamento de Psiquiatria, São Paulo, SP, Brazil.

**Keywords:** Sleep Initiation and Maintenance Disorders, Cognitive Behavioral Therapy, Hypnotics and Sedatives, Distúrbios do Início e da Manutenção do Sono, Terapia Cognitivo-Comportamental, Hipnóticos e Sedativo

## Abstract

Although, insomnia is one of the most common diseases that health professionals face in their practice, it receives little attention in medical training. Diagnosis is based on a careful history taking, and physicians must be aware of the diagnostic criteria. Insomnia should not be considered a symptom, but a comorbid condition. Although cognitive behavioral therapy (CBT) has been the mainstay treatment for insomnia for many years, it is usually regarded as a novel therapeutic strategy, both because of scarcity of qualified psychologists and of limited knowledge about insomnia among physicians. GABA receptor acting drugs are being abandoned in the treatment of insomnia because of abuse and dependence potential and accident risk. Two main current therapeutic options with the best scientific evidence are the tricyclic antidepressant, doxepin, and a new melatoninergic receptor agonist, ramelteon. Newer drugs to treat insomnia are in the pipeline. Hypocretine blocking agents will be marketed in the near future.

## RESUMO

Embora a insônia seja uma das doenças mais comuns encontrada por profissionais de saúde em sua prática quotidiana, está ainda é negligenciada nos currículos médicos. O diagnóstico baseia-se em anamnese cuidadosa e os médicos devem conhecer os critérios diagnósticos. A insônia não deve ser considerada apenas um sintoma, mas uma comorbidade. Apesar de a terapia cognitivo comportamental (TCC) para insônia ser a principal opção terapêutica há muito anos, esta modalidade terpêutica ainda é considerada uma nova estratégia pela escassez de psicólogos qualificados e pelo desconhecimento médico acerca da insônia. Os fármacos que atuam nos receptores GABA vêm sendo abandonados no tratamento da insônia devido ao potencial de abuso e dependência e pelo risco de acidentes. As duas principais opções terapêutica para insônia com melhor evidência científica são um antigo antidepressivo tricíclico, a doxepina, e um novo agonista do receptor melatoninérgico, a ramelteona. Novas drogas para insônia estão em processo de aprovação regulatória e comercialização. As mais próximas de serem liberadas para o mercado são as drogas bloqueadoras do sistema da hipocretina.

## INTRODUCTION

Although insomnia is a common and important problem in everybody’s life, it remains a neglected subject in medical training. Treatment of Insomnia may result in three different outcomes: great success, another medical failure or, worse, the beginning of long-term suffering and drug addiction.

 Therefore, physicians should be knowledgeable as to how to make a precise diagnosis, and also should master currently available treatment strategies to help patients achieve satisfactory therapeutic goals. 

## DIAGNOSIS OF INSOMNIA

Despite advances in neurology diagnostic tools, diagnosis of insomnia is largely based on skilled history taking. It is crucial that health professionals know the medical criteria to establish an accurate diagnosis of insomnia. It is also important that health care professionals understand that insomnia is not only a symptom, but it frequently is a comorbid condition related to other diseases, especially psychiatric disorders.

 The 3^rd^ edition of the International Classification of Sleep disorders (ICSD-3), published by the American Academy of Sleep Medicine^1^, and the 5^th^ edition of the Diagnostic and Statistical Manual of Mental Disorders (DSM-V), organized by the American Psychiatry Association^2^.share common aspects in the diagnostic criteria for insomnia Table 1. 

**Table 1.** Insomnia diagnostic criteria based on the ICSD-3 and the DSM-V.

Difficulty initiating, maintaining sleep, or waking up earlier than desired;
At least 3 nights/week of complain;
Acute insomnia is present for 3 months and chronic insomnia, for more than 3 months;
Sleep difficulty causes distress on social functioning or health. The main symptoms are listed below:
Fatigue
Attention, concentration, and memory impairment
Impairment in social, occupational, educational, or behavioural areas
Mood disturbance or irritability
Daytime sleepiness
Loss of motivation and initiative
Risk of accidents or errors
Concern about sleep or dissatisfaction
Patient must have adequate opportunity and circumstances to sleep
Sleep complain is not better explained by another sleep disorder
Insomnia complain is not adequately explained by coexisting mental disorders, medical conditions, or drug effect

 The idea that insomnia is not a symptom, but a comorbid condition related to medical and psychiatric diseases is due to the fact that insomnia may antecede other health conditions, and its persistence may increase the risk for recurrence of that condition. Additionally, insomnia usually requires a distinct therapeutic approach from the treatment for the underlying medical or psychiatric disorder^3^. In some circumstances, concomitant insomnia treatment may be the turning point for remission of the underlying disease. 

 Patients with insomnia are frequently erroneously ordered a polysomnography (PSG) exam. This is due to the fact that many physicians have the wrong concept that “if patients have a sleep problem, a sleep test will provide me with a diagnosis”. PSG is not needed for the diagnosis of insomnia, and PSG should only be ordered in this scenario if patients do not respond to treatment. PSG is indicated to investigate comorbid sleep disorders or to evaluate if there is a discrepancy between subjective and objective data^4^. If not correctly indicated, PSG in patients with insomnia will only result in a night with very poor sleep, and no additional useful information. 

 Likewise, actigraphy use in insomnia should be dictated to rule out comorbid sleep disorders, especially circadian rhythm disorders, and to evaluate sleep misperception. Actigraphy can possibly also be used to monitor treatment response^4^.

## TREATMENT OF INSOMNIA

Insomia treatment is based on two strategies that may be used isolatedly or in combination: cognitive behavioral therapy for insomnia (CBT-I) and pharmacological treatment. 

## CBT-I: AN “OLD NEW” STRATEGY AND PERSPECTIVES

CBT-I is considered the current main recommendation to treat insomnia: it shows good results, and few contraindications and side effects^5^. CBT-I shows the best long-term results in insomnia treatment^6^ ^-^ ^8^. Unfortunately, scarcity of skilled and qualified professionals limit the use of this therapeutic tool.

Difficult access contributed to the fact that CBT-I continues to be regarded as a novel strategy in insomnia treatment. CBT-I is performed by trained psychologists, and it is largely unknown in public and private mental health systems. CBT-I is not taught in most psychology courses in Brazil. An annual certification exam was established by the the Brazilian Sleep Association (Associação Brasileira do Sono) 2017, but availability of these professionals in the health system remains scarce, and CBT-I is usually offered as voluntary work in the public health system.

Despite all benefits, 19% to 26% of patients undergoing CBT-I do not obtain a satisfactory response^9^. Results are poorer if the patient presents psychiatric comorbidities^10^. Sleep restriction and stimulus control, the main CBT-I techniques, may cause transient discomfort, that may not be tolerated by some patients^11^ ^,^ ^12^. Therefore, newer approaches that may enhance efficacy of psychological treatment are being investigated.

### Mindfulness-based therapy for Insomnia (MBTI)

MBTI is a combination of mindfulness techniques and CBT-I, since mindfulness has shown good results in the treatment of insomnia^13^ ^,^ ^14^. While CBT-I challenges and substitutes dysfunctional thought contents, MBTI changes the relationship with these thoughts, promoting awareness and reducing cognitive hyperexcitement^15^. 

### Acceptance and commitment therapy based upon behavioural intervention for insomnia (ACT-BBI-I)

This strategy is also a complement to CBT-I using Acceptance and Commitment Therapy. ACT brings new perspectives to treat insomnia, because it is does not focus on symptoms, but increases behavioral and psychological flexibility to deal with symptoms, and with difficulties in adhering with CBT-I ^14^.

### Evaluation of personality traits response

Poor response to CBT-I is present in patients some personality traits^16^ ^-^ ^18^. Determining how these traits respond to different techniques may improve results of insomnia treatment. 

## PHARMACOLOGICAL TREATMENT

Current hypnotic drugs target neurotransmission in the ascending reticular activating system (ARAS), interrupting the wake signal. The main pharmacological effects of the currently available hypnotic drugs in Brazil are:

- Histamine 1 receptor antagonism;
- Serotonin 2A and 2C receptor agonism;
- Gamma-aminobutyric acid A receptor agonism - selective or not;
- Melatonin receptor agonism.

Additionally, noradrenergic, and acetyl-cholinergic antagonism also play a role, albeit of lesser importance, in the hypnotic effect. Gabapentinoid drugs, that inhibit voltage-dependent calcium channels are used off-label to treat insomnia. Hypocretin system antagonist drugs are available in other countries, with promising results.

## WHAT’S NEW ABOUT “OLD” HYPNOTICS?

Benzodiazepines (BZDs) were initially marketed in 1960. In 1963 diazepam was launched, and remained the main benzodiazepine for decades. Around 1977, BZDs became the most prescribed drug class in the world. BZDs also allowed physicians to decrease barbiturate use, with a safer pharmacological, profile^19^ BZD increases the effect of GABA_A_, the main inhibitory neurotransmitter system in the central nervous system (CNS).

With increasing knowledge about BZDs, it became clear that BZD side effects could harm patients, especially with longterm use and for the elderly. Abuse and dependence^20^, falls and fractures^21^ are well-established BZD related side effects. Association with dementia and mortality with BZD use has been described, but no cause-effect relationship has yet been established.

Since end of the 1990s, the new benzodiazepine receptor agonists (BzRA) have dominated the market for the pharmacological treatment of insomnia. In the following decade, these drugs were released in Brazil, represented by zolpidem, zopiclone and eszopiclone. These BzRAs act as selective GABA_A_ receptors on subunits α_1_ (zolpidem) and α_1_ + α_2_ (eszopiclone).

Expectations of lower risks of falls and fractures, abuse and dependence were largely unfulfilled^22^ ^,^ ^23^. Reports of car accidents caused a new FDA alert about the use of BzRA. Occurrences of disturbed behavior at emergency departments are also frequent^22^ ^-^ ^24^. There continues to be a lack of information regarding association of BzRAs and dementia.

BzRA are mostly used as sleep-inducing drugs, with an erroneous idea of “no risk”, leading to indiscriminate prescription. Currently the only formal indication for BzRA use is acute insomnia^4^. Most sleep specialists far more often discontinue BzRA, than prescribe, them.

Different strategies for BZDs and BzRA taper can be used, and CBT-I may be used as an adjuvant therapy, regardless of the pharmacological regimen. The use of safer hypnotics is usually recommended^4^ ^,^ ^21^.

Therefore, news about “old”, better GABA_A_ hypnotics, are not good. These drugs present risks that should be avoided, and restricted prescription must be the rule.

## WHAT ABOUT CURRENT HYPNOTIC DRUGS?

Current strategy for pharmacological treatment of insomnia in Brazil is based on a publication by the Brazilian Sleep Association (Associação Brasileira do Sono)^4^.

**Figure 1.**
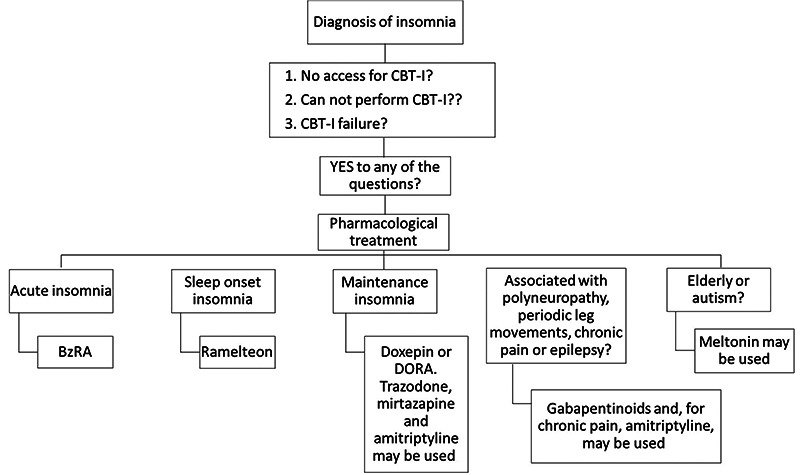
Flowchart for the treatment of insomnia.

Figure 1 depicts a flowchart with current strategies to treat insomnia with CBT-I and the decision to use pharmacological treatment. BzRA are only recommended for acute insomnia, as explained earlier. Distinction between sleep-onset insomnia and maintenance insomnia defines drug selection^4^ ^,^ ^25^ ^,^ ^26^.

Amitriptyline, mirtazapine, trazodone, and gabapentinoids are familiar drugs to most neurologists. Two less known drugs (ramelteon and doxepin) will be discussed here. Since suvorexant and other hypocretin receptor agonists are not yet available in Brazil, these drugs will be only briefly discussed.

### Ramelteon

Ramelteon is a melatonin 1 (MT_1_) and 2 (MT_2_) receptor agonist, with higher affinity with MT_1_ than melatonin. Ramelteon is absorbed rapidly, with median peak concentrations at approximately 0.75 hour (0.5 to 1.5 hours) after oral administration on an empty stomach. Its main metabolite has a half-life of two to five hours, independent of doses. The recommended dosage is 8mg, not to be increased. Patients should take it orally 30 minutes before going to bed. Most common adverse effects include dizziness, somnolence, fatigue, headache, dysgeusia and nausea^27^ ^,^ ^28^.

### Doxepin

Doxepin is a tricyclic antidepressant with much higher affinity to H_1_ receptors (antagonistic) than to serotoninergic, noradrenergic, and cholinergic receptors. Time to peak plasma concentration is around two hours, and half-life ranges from six to eight hours. Patients should take it orally around two hours before going to bed. Antidepressant doses are between 100mg and 300mg. Doses for insomnia range from 1mg to 6mg, usually starting at 3mg. Adverse effects are similar to those seen with other tricyclic antidepressants. Due to doxepin affinity to H_1_ receptors, main complaints are related to antihistaminergic effects (somnolence, fatigue, weakness, lethargy)^28^ ^,^ ^29^.

In Brazil, there are no commercial formulations of doxepin, and patients may obtain this medication in formulation pharmacies.

## WHAT’S IN THE FUTURE OF INSOMNIA TREATMENT?

### Dual orexinergic receptor antagonists

Promising novel insomnia pharmacological treatment agents act as hypocretin receptor antagonists, also known as DORA (dual orexinergic receptor antagonists). Suvorexant and lemborexant are commercially available in other countries, but still not in Brazil.

 These drugs block both hypocretinergic system receptors - HcrtR_1_ and HcrtR_2_ - and, consequently, interrupt ARAS activation, promoting sleep maintenance.

 Suvorexant doses range from 10mg to 20mg a day. Peak plasma time is two hours, ranging but between 0.5 and six hours. Suvorexant half-life is around 12 hours. Lemborexant can be used in two doses: 5mg and 10mg. Peak plasma concentration ranges from one to three hours, with and a half-life of 17 hours with 5mg, and 19 hours with 10mg.

 The main difficulty of managing this drug class is due to drug-to-drug interaction, increasing effect of other CNS depressors .Adverse efeects are similar to other hypnotics: somnolence, fatigue, and headache. Side effects related to hypocretin antagonism can be seen: sleep paralysis, hallucinations, nightmares or abnormal dreams^28^ ^,^ ^30^. 

A new DORA, daridorexant, is close to being released. Daridorexant is expected to cause lower daytime somnolence because of a shorter half-life of eight hours^31^. A selective HcrtR_2_ antagonist, seltorexant, is also under development, possibly with lower peak plasma concentration and half-life^32^.

### Promises for insomnia pharmacological treatment

 Other drug mechanisms that may promote sleep are currently being evaluated:

- GABA_A_ receptor enhancer^30^ ^,^ ^33^;
- Selective^30^ ^,^ ^33^; (NÃO FAZ SENTIDO)
- Combined melatonin and serotonin-receptor agonists^30^ ^,^ ^33^;
- Histamine receptor inverse agonist^30^ ^,^ ^33^;
- Cannabinoid formulations^34^.

GABA_A_ receptor enhancers appear to be more effective to promote and maintain sleep. The goal is to develop a drug that does not cause the feared side effects.

 Pharmaceutical companies have withdrawn selective 2A serotonin receptor and histamine inverse agonists. These remain as possible targets to be explored.

 A promising melatonin and serotonin 5A receptor agonist, piromelatine, was studied in a phase II study, and is currently being evaluated for Alzheimers disease.

 Cannabinoid formulations are a promising drug class in the treatment of insomnia, but pharmacology is still poorly understood. There is no formal indication for the use of cannabinoid formulations in insomnia but, unfortunately, economic interests are dictating the practice of cannabinoid use for insomnia. Crucial questions left to be answered: What disease? Which patient? What doses? What are yhe adverse effects? What are the drug interactions? 

In conclusion, despite its high prevalence in the general population, insomnia is still a neglected disease in the training of health professionals and clinical practice after graduation is mostly the only way physicians learn how to manage insomnia. Insomnia represents a population with risk of long-term insomnia or of adverse effects of drugs.

 There are different strategies to manage insomnia, and neurologists must be familiar with both non-pharmacological and pharmacological treatments options, to prescribe the therapeutic regimen that best fits each patient.

## References

[B1] Medicine American Academy of Sleep Medicine (2014). International classification of sleep disorders.

[B2] American Psychiatric Association (2013). Diagnostic and statistical manual of mental disorders.

[B3] NHI (2005). NIH State-of-the-Science Conference Statement on Manifestations and Management Office of the Director. NIH Consens State Sci Statements.

[B4] Bacelar A, Pinto LR (2019). Insônia: do diagnóstico ao tratamento.

[B5] Edinger JD, Arnedt JT, Bertisch SM, Carney CE, Harrington JJ, Lichstein KL (2021). Behavioral and psychological treatments for chronic insomnia disorder in adults: an American Academy of Sleep Medicine clinical practice guideline. J Clin Sleep Med.

[B6] Sivertsen B, Omvik S, Pallesen S, Bjorvatn B, Havik OE, Kvale G (2006). Cognitive behavioral therapy vs zopiclone for treatment of chronic primary insomnia in older adults: a randomized controlled trial. JAMA.

[B7] Jacobs GD, Pace-Schott EF, Stickgold R, Otto MW (2004). Cognitive behavior therapy and pharmacotherapy for insomnia: a randomized controlled trial and direct comparison. Arch Intern Med.

[B8] Morin CM, Colecchi C, Stone J, Sood R, Brink D (1999). Behavioral and pharmacological therapies for late-life insomnia: a randomized controlled trial. JAMA.

[B9] Harvey AG, Tang NKY (2003). Cognitive behaviour therapy for primary insomnia: can we rest yet?. Sleep Med Rev.

[B10] Wu JQ, Appleman ER, Salazar RD, Ong JC (2015). Cognitive behavioral therapy for insomnia comorbid with psychiatric and medical conditions: a meta-analysis. JAMA Intern Med.

[B11] Riedel BW, Lichstein KL (2001). Strategies for evaluating adherence to sleep restriction treatment for insomnia. Behav Res Ther.

[B12] Vincent NK, Hameed H (2003). Relation between adherence and outcome in the group treatment of insomnia. Behav Sleep Med.

[B13] Ong JC, Manber R, Segal Z, Xia Y, Shapiro S, Wyatt JK (2014). A randomized controlled trial of mindfulness meditation for chronic insomnia. Sleep.

[B14] Ong JC, Shapiro SL, Manber R (2009). Mindfulness meditation and cognitive behavioral therapy for insomnia: a naturalistic 12-month follow-up.

[B15] Ong JC, Smith CE (2017). Using mindfulness for the treatment of insomnia. Curr Sleep Med Rep.

[B16] Gurtman CG, Mcnicol R, Mcgillivray JA (2014). The role of neuroticism in insomnia. Clin Psychol.

[B17] Bliwise DL, Friedman L, Nekich JC, Yesavage JA (1995). Prediction of outcome in behaviorally based insomnia treatments. J Behav Ther Exp Psychiatry.

[B18] Johann AF, Riemann D, Spiegelhalder K (2018). Does perfectionism increase the risk for dropout from cognitive behavioral therapy for insomnia?. J Clin Sleep Med.

[B19] Balon R, Starcevic V, Silberman E, Cosci F, Dubovsky S, Fava GA (2020). The rise and fall and rise of benzodiazepines: a return of the stigmatized and repressed. Braz J Psychiatry.

[B20] O'Brien CP (2005). Benzodiazepine use, abuse, and dependence. J Clin Psychiatry.

[B21] Markota M, Rummans TA, Bostwick JM, Lapid MI (2016). Benzodiazepine use in older adults: dangers, management, and alternative therapies. Mayo Clin Proc.

[B22] Gunja N (2013). The clinical and forensic toxicology of Z-drugs. J Med Toxicol.

[B23] Brandt J, Leong C (2017). Benzodiazepines and Z-Drugs: an updated review of major adverse outcomes reported on in epidemiologic research. Drugs R D.

[B24] U.S. Food and Drug Administration (2013). Risk of next‐morning impairment after use of insomnia drugs; FDA requires lower recommended doses for certain drugs containing zolpidem (Ambien, Ambien CR, Edluar, and Zolpimist). Saf Accouncement.

[B25] Sateia MJ, Buysse DJ, Krystal AD, Neubauer DN, Heald JL (2017). Clinical practice guideline for the pharmacologic treatment of chronic insomnia in adults: an American Academy of Sleep Medicine Clinical Practice Guideline. J Clin Sleep Med.

[B26] Riemann D, Baglioni C, Bassetti C, Bjorvatn B, Groselj LD, Ellis JG (2017). European guideline for the diagnosis and treatment of insomnia. J Sleep Res.

[B27] Mcgechan A, Wellington K. Ramelteon (2005). Ramelteon. CNS Drugs.

[B28] Atkin T, Comai S, Gobbi G (2018). Drugs for insomnia beyond benzodiazepines: pharmacology, clinical applications, and discovery. Pharmacol Rev.

[B29] Smaglik P (2003). New perspectives. Nature.

[B30] Abad VC, Guilleminault C (2018). Insomnia in elderly patients: recommendations for pharmacological management. Drugs Aging.

[B31] Roch C, Bergamini G, Steiner MA, Clozel M (2021). Nonclinical pharmacology of daridorexant: a new dual orexin receptor antagonist for the treatment of insomnia. Psychopharmacology (Berl).

[B32] Recourt K, de Boer P, Zuiker R, Luthringer R, Kent J, van der Ark P (2019). The selective orexin-2 antagonist seltorexant (JNJ-42847922/MIN-202) shows antidepressant and sleep-promoting effects in patients with major depressive disorder. Transl Psychiatry.

[B33] Zisapel N (2015). Current phase II investigational therapies for insomnia. Expert Opin Investig Drugs.

[B34] Kesner AJ, Lovinger DM (2020). Cannabinoids, endocannabinoids and sleep.

